# Effects of different salt sources and salinity levels on emergence and seedling growth of faba bean genotypes

**DOI:** 10.1038/s41598-021-97810-6

**Published:** 2021-09-14

**Authors:** Nurlykhan Bimurzayev, Hatice Sari, Ahmet Kurunc, Kıvanc Hayri Doganay, Mulat Asmamaw

**Affiliations:** 1 Department of Agricultural Structures and Irrigation, Faculty of Agriculture, Akdeniz University, Campus, 07058 Antalya, Turkey; 2 Department of Field Crops, Faculty of Agriculture, Akdeniz University, Campus, 07058 Antalya, Turkey

**Keywords:** Physiology, Plant sciences

## Abstract

Suitability of poor quality water for irrigation depends on salinity level and solute concentration in the water and selected crop. Salt stress is a major potential constraint for faba bean. The present study aimed to investigate the effects of different Cl- and SO_4_-containing salt sources in irrigation water with different salinity levels on emergence, early seedling growth and photosynthetic capacity of six faba bean genotypes. The negative effect order of salinity level was high (3 dS/m) > medium (2 dS/m) > low (1 dS/m) > control (0.05 dS/m) for all investigated parameters except dry root weight. The negative effects of Cl-containing salt sources were higher than that of SO_4_-containing salt sources. The worst and the best performing genotypes were determined as III-28 and III-29 on emergence percentage at 10th DAS, I-29 and III-1 on mean emergence time, III-22 and III-1 on shoot height, III-1 and I-29 on fresh biomass weight, III-22 and III-28 on fresh shoot weight, III-29 and I-29 on fresh root weight, respectively. This study showed that faba bean genotypes have different behaviors in terms of response to the increasing salinity levels artificially makeup by using different salt sources indicating that salt response of faba bean is genotype-specific.

## Introduction

Faba bean (*Vicia faba* L.) also referred to as broad bean, horse bean or field bean, is a good source of protein, energy and fiber and the crop is widely grown for food and feed^1^. It is used as a source of protein in human diets, as fodder and a forage crop for animals, and for its excellent ability to fix atmospheric nitrogen. Seeds of faba bean are consumed in large quantities in developing countries such as China, Turkey, Egypt, Ethiopia, and Central America^2^. In addition to a good alternative to animal proteins, it is also an attractive product because of its low cost, long storage life, and easy transportation^3^. In terms of world production, faba bean ranked sixth with 4.5 Mt from 2.5 Mha after common bean (*Phaseolus vulgaris* L.), pea (*Pisum sativum* L.), chickpea (*Cicer arietinum* L.), cowpea (*Vigna unguiculata* L. Walp.), and lentil (*Lens culinaris* Medik.)^4^. Faba bean is the fourth most widely grown pulse crop in Turkey^5^. In Turkey, the cultivated area of faba bean seeds for food and feed are 2312 and 2020 ha in 2019 with the average yields of 2.37 and 3.40 tons per hectare and with total productions of 5484 and 6862 tons, respectively^6^.

All agricultural soils and irrigation water contain mineral salts. The amount and kind of salt present depends on the makeup of both the soil and irrigation water^7^. The ionic composition of irrigation water is as important as the level of water salinity. Each type of salt in the soil and irrigation water may exert different effects on plant growth^8^. Particularly in the semi-arid regions of the world, such as the Mediterranean Sea, the supply of sufficient quality water for irrigation is an important problem. This situation is leading to the use of groundwater containing excessive amounts of soluble salts with chlorine compounds in the coastal areas^9^. In contrast, the water sources in the inner parts contains sulfide compounds. It was reported that electrical conductivity values of groundwater used for irrigation purposes in greenhouses in Antalya, ranged from 0.85 to 4.1 dS/m in November (winter) to 0.83–4.4 dS/m in June (summer)^10^.

Use of poor quality or saline water for irrigation is a subject of increasing interest for sustaining crop productivity. Although salinity affects the crops at all growth stages of development, plant sensitivity to salinity for most crops varies from one stage to the next. Most of the salinity effect on crops were obtained from salinity treatments imposed after seedlings were established in non-saline plots and do not necessarily apply to germination, emergence and early seedling stages^11,12^. Salinity is also known to impair seed germination, emergence and early seedling growth. In addition, in many studies on salt tolerance, it is not been possible to determine the detrimental effects on growth that is associated with whether the level of salinity or the kind of salt present in the water or soil solution. Therefore, the exploitation of seed performance under salt and salinity effect on emergence and early seedling growth stage will be helpful for improving the choice of genotype suited to perform best under known conditions of salinity^13^.

Faba bean has a bright future as a protein crop that provides additional crop rotation benefits for many areas of the world. However, salinity is an ever-present major constraint and a major threat to these kind of legume crops, particularly in areas with irrigated agriculture^14^. This plant is known to be moderately sensitive to salinity and should, therefore, be cultivated on soils with little or no saline content^11^. The harmful effects have been reported for faba bean genotypes under saline conditions composed of NaCl^15–17^. However, no information is available regarding the comparison of CaCl_2_, MgCl_2_, CaSO_4_, MgSO_4_ and Na_2_SO_4_ salts and salinity levels. Ayers and Westcot^12^ claim that the suitability of water for irrigation should be determined by the amount and kind of salt present. Also, emergence percentage and early growth of crops under a particular level of salt vary considerably among genotypes^18^. Therefore, the present study aimed to evaluate the effects of different salt sources and irrigation water salinity levels on the emergence and early growth parameters of six faba bean genotypes.

## Results

The results of ANOVA for faba bean genotypes (G), salt sources (S) and salinity levels (L) as the main factors, and their interactions including G × S, G × L, S × L and G × S × L were given in Table 1. According to these statistical analyses, faba bean genotypes and salinity level as the main factors and S × L interaction had significant effects on all investigated parameters including emergence percentage at 10th, 17th (*P* < 0.05 for S × L) and 24th (*P* < 0.05 for S × L) DAS, mean emergence time; shoot height; fresh and dry biomass, shoot and root weights; and F_v_/F_m_ ratio. However, emergence percentage at 17th and 24th DAS for salt sources as the main effect; emergence percentage at 17th DAS, mean emergence time, dry shoot weight and F_v_/F_m_ ratio for G × S interaction; and only F_v_/F_m_ ratio for G × L interaction were not statistically significant (Table 1). Since a three-way ANOVA did not show significant differences for all parameters (except fresh root weight), the results were interpreted by following two-way interaction of S × L for each faba bean genotype and also by plotting S × G and L × G interactions by ignoring salinity level and salt sources, respectively^19^. Therefore, the performances of each faba bean genotype under S × L interaction, and individual effect of salinity levels averaged over salt sources and salt sources averaged over salinity level for all faba bean genotypes were evaluated.

**Table 1:** *P* values (significance) from analysis of variance for the effects of faba bean genotype, salt source and salinity level on investigated parameters.

Source of variation	Mean square						
	Emergence percentage @ DAS			Mean Emergence Time	Shoot height	Biomass weight (g)	
	10th	17th	24th	(day)	(cm)	Fresh	Dry
Genotype (G)	< 0.001	< 0.001	< 0.001	< 0.001	< 0.001	< 0.001	< 0.001
Salt source (S)	< 0.001	0.152	0.602	< 0.001	< 0.001	< 0.001	< 0.001
Salinity level (L)	< 0.001	< 0.001	0.002	< 0.001	< 0.001	< 0.001	< 0.001
G × S	0.024	0.112	0.041	0.208	< 0.001	< 0.001	0.002
G × L	< 0.001	< 0.001	0.002	< 0.001	< 0.001	< 0.001	< 0.001
S × L	< 0.001	0.030	0.046	< 0.001	< 0.001	< 0.001	< 0.001
G × S × L	0.211	0.597	0.373	0.677	0.189	0.101	0.802

Source of variation	Mean square						
	Shoot weight (g)		Root weight (g)		F_v_/F_m_		
	Fresh	Dry	Fresh	Dry			
Genotype (G)	< 0.001	< 0.001	< 0.001	< 0.001	< 0.001		
Salt source (S)	< 0.001	< 0.001	< 0.001	< 0.001	< 0.001		
Salinity level (L)	< 0.001	< 0.001	< 0.001	< 0.001	< 0.001		
G × S	0.009	0.349	< 0.001	< 0.001	0.081		
G × L	< 0.001	< 0.001	< 0.001	< 0.001	0.616		
S × L	< 0.001	< 0.001	< 0.001	< 0.001	< 0.001		
G × S × L	0.341	0.746	0.005	0.225	0.332		

### Performance of I-29 faba bean genotype

Considering S × L interaction, F_v_/F_m_ ratios showed a significant difference for I-29 genotype, whereas emergence percentage at 10th (ranged between 0.0 and 26.7%), and at 24th (ranged between 63.3 and 100.0%) DAS, mean emergence time (ranged between 11.47 and 15.77 days), shoot height (ranged between 4.51 and 9.23 cm), fresh biomass weight (ranged between 6.73 and 12.40 g), dry biomass weight (ranged between 1.03 and 1.51 g), fresh shoot weight (ranged between 2.35 and 5.40 g), dry shoot weight (ranged between 0.31 and 0.71 g), fresh root weight (ranged between 4.38 and 7.56 g) and dry root weight (ranged between 0.56 and 0.92 g) parameters were not affected from S × L interaction (Table 2). None of the emergence percentage values starting from on the 9th DAS until the experiment lasted was not statistically significant. As an indication of photosynthetic capacity, the lowest F_v_/F_m_ ratio was observed under low, medium and high salinity levels with Na_2_SO_4_ salt source, however F_v_/F_m_ ratios under all salinity levels with other salt sources did not show significant difference from that of the control salinity level (Table 2).

**Table 2:** The interaction effects of S × L on I-29 faba bean genotype.

Parameter	Salinity level (dS/m)	Salt source					
		CaCl_2_	MgCl_2_	NaCl	CaSO_4_	MgSO_4_	Na_2_SO_4_
Emergence percentage at 10th DAS	0.0	20.00^x^a^y^	20.00a	20.00a	20.00a	20.00a	20.00a
	1.0	0.00a	23.33a	26.67a	20.00a	26.67a	10.00a
	2.0	10.00a	3.33a	6.66a	20.00a	30.00a	16.67a
	3.0	0.00a	3.33a	3.33a	20.00a	16.67a	26.67a
Emergence percentage at 24th DAS	0.0	93.33a	93.33a	93.33a	93.33a	93.33a	93.33a
	1.0	93.33a	76.67a	90.00a	93.33a	90.00a	86.67a
	2.0	90.00a	76.67a	80.00a	83.33a	100.00a	86.67a
	3.0	63.33a	70.00a	73.33a	76.67a	90.00a	83.33a
Mean emergence time (day)	0.0	12.00a	12.00a	12.00a	12.00a	12.00a	12.00a
	1.0	11.56a	11.98a	12.22a	11.53a	11.47a	12.92a
	2.0	12.98a	13.12a	13.84a	12.17a	11.70a	11.89a
	3.0	15.77a	15.28a	13.08a	13.42a	12.81a	12.60a
Shoot height (cm)	0.0	9.23a	9.23a	9.23a	9.23a	9.23a	9.23a
	1.0	8.61a	7.08a	8.12a	6.65a	8.39a	6.60a
	2.0	6.46a	5.05a	5.52a	7.45a	6.97a	7.29a
	3.0	4.55a	4.81a	4.51a	6.58a	5.67a	4.51a
Fresh biomass weight (g)	0.0	11.17a	11.17a	11.17a	11.17a	11.17a	11.17a
	1.0	12.40a	10.20a	12.02a	10.13a	11.26a	10.22a
	2.0	9.35a	7.95a	8.47a	11.18a	9.56a	10.95a
	3.0	7.75a	6.73a	8.22a	11.79a	7.41a	7.12a
Dry biomass weight (g)	0.0	1.39a	1.39a	1.39a	1.39a	1.39a	1.39a
	1.0	1.39a	1.15a	1.40a	1.17a	1.41a	1.34a
	2.0	1.17a	1.21a	1.03a	1.19a	1.48a	1.51a
	3.0	1.03a	1.20a	1.12a	1.42a	1.37a	1.13a
Fresh shoot weight (g)	0.0	4.60a	4.60a	4.60a	4.60a	4.60a	4.60a
	1.0	5.40a	4.41a	4.77a	3.78a	4.74a	4.14a
	2.0	4.20a	3.22a	3.53a	4.52a	4.09a	4.35a
	3.0	2.59a	2.35a	2.97a	4.24a	2.91a	2.53a
Dry shoot weight (g)	0.0	0.62a	0.62a	0.62a	0.62a	0.62a	0.62a
	1.0	0.66a	0.54a	0.66a	0.54a	0.71a	0.60a
	2.0	0.61a	0.50a	0.38a	0.59a	0.60a	0.59a
	3.0	0.31a	0.42a	0.47a	0.61a	0.49a	0.39a
Fresh root weight (g)	0.0	6.56a	6.56a	6.56a	6.56a	6.56a	6.56a
	1.0	7.00a	5.79a	7.25a	6.35a	6.52a	6.07a
	2.0	5.15a	4.72a	4.93a	6.65a	5.46a	6.60a
	3.0	5.16a	4.38a	5.26a	7.56a	4.50a	4.59a
Dry root weight (g)	0.0	0.77a	0.77a	0.77a	0.77a	0.77a	0.77a
	1.0	0.73a	0.60a	0.74a	0.62a	0.71a	0.74a
	2.0	0.56a	0.71a	0.65a	0.60a	0.88a	0.92a
	3.0	0.71a	0.78a	0.65a	0.81a	0.88a	0.74a
F_v_/F_m_	0.0	0.67abc	0.67abc	0.67abc	0.67abc	0.67abc	0.67abc
	1.0	0.67abc	0.65bcd	0.64cde	0.68ab	0.71a	0.60d
	2.0	0.66bcd	0.66bcd	0.68ab	0.68ab	0.68ab	0.62d
	3.0	0.66bcd	0.65bcd	0.66bcd	0.71a	0.64cde	0.55f

^x^Each value is the mean of three replications.

^y^Within salt source and salinity level of each parameter, means followed by the same letter are not significantly different at the 5% level according to Duncan’s multiple range tests.

### Performance of III-1 faba bean genotype

Emergence percentage at 24th DAS, mean emergence time and dry root wieght of the III-1 faba bean genotype were ranged from 70.0 to 100.0%, from 9.31 to 12.69 days and from 0.40 to 0.68 g, respectively, but there was no statistically significant difference among these values under S × L interaction. Throughout the experimental period, only the emergence percentage at 9th and 10th DAS showed significant difference. Shoot height of this genotype were significantly decreased under each increasing salinity levels with all salt sources. In general, medium and high salinity levels with CaCl_2_ and Na_2_SO_4_, and high salinity level with MgCl_2_, NaCl and MgSO_4_ salt sources caused progressive decreases on fresh biomass, shoot and root weight whereas no effect of the CaSO_4_ salt under all salinity level was observed on these parameters. Similarly, medium and high salinity levels with CaCl_2_ and high salinity level with Na containing salts; and medium and high salinity levels with CaCl_2_, MgCl_2_ and Na_2_SO_4_, and high salinity level with NaCl salt source caused significant reductions on dry biomass and shoot weights, respectively. However, both parameter values under all salinity levels with CaSO_4_ and MgSO_4_ in addition to MgCl_2_ (only for dry biomass weights) salt sources did not show statistical significance. Profoundly low F_v_/F_m_ ratios were observed under medium and high salinity levels with Na_2_SO_4_ salt source (Table 3).

**Table 3:** The interaction effects of S × L on III-1 faba bean genotype.

Parameter	Salinity level (dS/m)	Salt source					
		CaCl_2_	MgCl_2_	NaCl	CaSO_4_	MgSO_4_	Na_2_SO_4_
Emergence percentage at 10th DAS	0.0	73.33^x^ab^y^	73.33ab	73.33ab	73.33ab	73.33ab	73.33ab
	1.0	46.67bcdef	56.67abcde	76.67ab	63.33abc	63.33abc	83.33a
	2.0	26.67fg	33.33efg	33.33efg	73.33ab	60.00abcd	43.33cdefg
	3.0	20.00g	26.67fg	36.67defg	73.33ab	36.67defg	56.67abcde
Emergence percentage at 24th DAS	0.0	86.67a	86.67a	86.67a	86.67a	86.67a	86.67a
	1.0	90.00a	96.67a	100.00a	90.00a	80.00a	86.67a
	2.0	90.00a	96.67a	86.67a	83.33a	80.00a	73.33a
	3.0	83.33a	70.00a	90.00a	90.00a	73.33a	86.67a
Mean emergence time (day)	0.0	9.65a	9.65a	9.65a	9.65a	9.65a	9.65a
	1.0	11.37a	10.68a	10.77a	10.29a	10.25a	9.31a
	2.0	11.78a	11.14a	11.47a	9.54a	11.01a	10.15a
	3.0	12.69a	11.18a	11.71a	10.31a	11.02a	10.14a
Shoot height (cm)	0.0	10.75a	10.75a	10.75a	10.75a	10.75a	10.75a
	1.0	7.58bcd	7.06cde	7.63bcd	8.12bcd	8.49bc	8.01bcd
	2.0	4.34fg	6.55de	4.92fg	8.32bc	7.41bcd	5.75ef
	3.0	4.00g	3.98g	3.88g	8.76b	6.67de	4.57fg
Fresh biomass weight (g)	0.0	9.66abc	9.66abc	9.66abc	9.66abc	9.66abc	9.66abc
	1.0	9.05abc	8.75abcd	8.87abcd	9.43abc	10.46ab	7.86bcde
	2.0	6.17de	8.22bcde	6.93cde	11.62a	7.12cde	5.58e
	3.0	6.06de	5.54e	5.79e	11.51a	5.67e	5.54e
Dry biomass weight (g)	0.0	1.13abcd	1.13abcd	1.13abcd	1.13abcd	1.13abcd	1.13abcd
	1.0	0.91cde	1.02bcde	0.89cde	1.02bcde	1.33a	1.13abcd
	2.0	0.79e	0.87cde	0.96cde	1.27ab	1.16abc	0.87cde
	3.0	0.74e	0.84de	0.78e	1.27ab	1.11abcd	0.80e
Fresh shoot weight (g)	0.0	4.44ab	4.44ab	4.44ab	4.44ab	4.44ab	4.44ab
	1.0	4.16abc	4.07abc	3.85abc	3.97abc	4.68a	3.98abc
	2.0	2.74d	3.94abc	3.08cd	4.45ab	3.35bcd	2.49d
	3.0	2.60d	2.25d	2.32d	4.89a	2.59d	2.43d
Dry shoot weight (g)	0.0	0.59ab	0.59ab	0.59ab	0.59ab	0.59ab	0.59ab
	1.0	0.52abcd	0.55abc	0.50abcde	0.50abcde	0.65a	0.56abc
	2.0	0.37ef	0.46cdef	0.50abcde	0.65a	0.52abcd	0.40def
	3.0	0.32g	0.34fg	0.35fg	0.66a	0.48bcdef	0.37ef
Fresh root weight (g)	0.0	5.23abcd	5.23abcd	5.23abcd	5.23abcd	5.23abcd	5.23abcd
	1.0	4.89bcdef	4.69bcdef	5.02bcdef	5.46abcd	5.78abc	3.88cdef
	2.0	3.43ef	4.28cdef	3.85cdef	7.16a	3.77def	3.09f
	3.0	3.46ef	3.30ef	3.47ef	6.62ab	3.08f	3.12f
Dry root weight (g)	0.0	0.54a	0.54a	0.54a	0.54a	0.54a	0.54a
	1.0	0.40a	0.46a	0.40a	0.52a	0.68a	0.57a
	2.0	0.41a	0.41a	0.47a	0.61a	0.64a	0.48a
	3.0	0.41a	0.50a	0.43a	0.61a	0.62a	0.43a
F_v_/F_m_	0.0	0.66bcde	0.66bcde	0.66bcde	0.66bcde	0.66bcde	0.66bcde
	1.0	0.65bcde	0.67abcd	0.65bcde	0.68abc	0.70a	0.61ef
	2.0	0.66bcde	0.68abc	0.66bcde	0.66bcde	0.69ab	0.57g
	3.0	0.69ab	0.63cde	0.65bcde	0.69ab	0.62def	0.59fg

^x^Each value is the mean of three replications.

^y^Within salt source and salinity level of each parameter, means followed by the same letter are not significantly different at the 5% level according to Duncan’s multiple range tests.

### Performance of III-22 faba bean genotype

Although emergence percentage at 10th DAS, shoot height, fresh shoot weight and dry root weight values were ranged between 0.0 and 30.0%, 3.60 and 8.17 cm, 2.01 and 4.23 g and 0.47 and 0.71 g, respectively, there was no significant difference among these values under S × L interaction. Emergence percentage values after 12th DAS were significantly different. The lowest emergence percentage at 24th DAS was observed under high salinity level with NaCl salt source. The statistical results revealed that high salinity level with CaCl_2_, medium and high salinity levels with NaCl salt sources significantly retarded the mean emergence time of III-2 seeds. Compared to control treatment, progresive decreases on fresh biomass weight under high salinity level with CaCl_2_, and NaCl, and under medium and high salinity levels with MgCl_2_ salt sources; on dry biomass weight under high salinity level with Na containing salts; on dry shoot weight under high salinity level with all Cl containing salts; and on fresh root weight under high salinity level with CaCl_2_ and medium and high salinity levels with MgCl_2_ and NaCl salt sources. Similar to III-1, slightly low F_v_/F_m_ ratios were observed under medium and high salinity levels with Na_2_SO_4_ salt source (Table 4).

**Table 4:** The interaction effects of S × L on III-22 faba bean genotype.

Parameter	Salinity level (dS/m)	Salt source					
		CaCl_2_	MgCl_2_	NaCl	CaSO_4_	MgSO_4_	Na_2_SO_4_
Emergence percentage at 10th DAS	0.0	16.67^x^a^y^	16.67a	16.67a	16.67a	16.67a	16.67a
	1.0	16.67a	26.67a	6.67a	6.67a	23.33a	30.00a
	2.0	6.67a	6.67a	0.00a	13.33a	26.67a	30.00a
	3.0	0.00a	6.67a	0.00a	16.67a	20.00a	30.00a
Emergence percentage at 24th DAS	0.0	96.67ab	96.67ab	96.67ab	96.67ab	96.67ab	96.67ab
	1.0	93.33ab	96.67ab	93.33ab	93.33ab	96.67ab	100.00a
	2.0	100.00a	86.67bc	100.00a	93.33ab	90.00abc	100.00a
	3.0	90.00abc	100.00a	80.00c	90.00abc	100.00a	100.00a
Mean emergence time (day)	0.0	11.79cde	11.79cde	11.79cde	11.79cde	11.79cde	11.79cde
	1.0	12.57bcd	11.51cde	12.62bcd	11.97bcde	11.83cde	11.57cde
	2.0	12.10bcde	12.19bcde	13.30ab	11.97bcde	11.75cde	11.30de
	3.0	14.17a	12.87bc	14.25a	12.48bcd	11.00e	11.27de
Shoot height (cm)	0.0	8.17a	8.17a	8.17a	8.17a	8.17a	8.17a
	1.0	7.01a	6.69a	6.24a	5.57a	6.68a	6.70a
	2.0	5.70a	5.61a	5.23a	6.40a	6.47a	6.13a
	3.0	4.38a	4.27a	3.60a	5.67a	5.73a	4.70a
Fresh biomass weight (g)	0.0	8.46cd	8.46cd	8.46cd	8.46cd	8.46cd	8.46cd
	1.0	9.05abcd	10.51a	9.65abc	8.20cd	9.06abcd	10.16ab
	2.0	8.28cd	6.61ef	7.56de	8.93bcd	9.68abc	10.31ab
	3.0	5.76fg	5.76fg	4.43g	8.05d	8.42cd	8.35cd
Dry biomass weight (g)	0.0	1.16bcd	1.16bcd	1.16bcd	1.16bcd	1.16bcd	1.16bcd
	1.0	1.19ab	1.37a	1.16bcd	1.17bc	1.16bcd	1.13bcd
	2.0	1.10bcde	1.11bcde	0.99cdef	1.22ab	1.24ab	1.12bcde
	3.0	0.96def	0.98cdef	0.85f	1.22ab	1.12bcde	0.93ef
Fresh shoot weight (g)	0.0	3.66a	3.66a	3.66a	3.66a	3.66a	3.66a
	1.0	4.21a	4.11a	3.83a	3.54a	3.89a	4.23a
	2.0	3.68a	3.08a	3.54a	4.01a	4.03a	4.17a
	3.0	2.47a	2.60a	2.01a	3.20a	3.27a	3.19a
Dry shoot weight (g)	0.0	0.51bcde	0.51bcde	0.51bcde	0.51bcde	0.51bcde	0.51bcde
	1.0	0.56abcd	0.66a	0.54abcd	0.54abcd	0.57abcd	0.54abcd
	2.0	0.48cdef	0.55abcd	0.45def	0.63ab	0.65ab	0.60abc
	3.0	0.37fg	0.38fg	0.32g	0.54abcd	0.55abcd	0.45def
Fresh root weight (g)	0.0	4.81de	4.81de	4.81de	4.81de	4.81de	4.81de
	1.0	4.84de	6.40a	5.82ab	4.65de	5.17bcd	5.93ab
	2.0	4.60de	3.53fg	4.02ef	4.92cd	5.65abc	6.13a
	3.0	3.28fg	3.16g	2.42h	4.85de	5.15bcd	5.16bcd
Dry root weight (g)	0.0	0.65a	0.65a	0.65a	0.65a	0.65a	0.65a
	1.0	0.63a	0.71a	0.62a	0.63a	0.58a	0.60a
	2.0	0.61a	0.55a	0.53a	0.59a	0.59a	0.52a
	3.0	0.59a	0.60a	0.52a	0.68a	0.57a	0.47a
F_v_/F_m_	0.0	0.66bcd	0.66bcd	0.66bcd	0.66bcd	0.66bcd	0.66bcd
	1.0	0.67abc	0.66bcd	0.64cde	0.68abc	0.70a	0.63def
	2.0	0.67abc	0.67abc	0.65cde	0.66bcd	0.68abc	0.61ef
	3.0	0.66bcd	0.65cde	0.65cde	0.70a	0.63def	0.60f

^x^Each value is the mean of three replications.

^y^Within salt source and salinity level of each parameter, means followed by the same letter are not significantly different at the 5% level according to Duncan’s multiple range tests.

### Performance of III-28 faba bean genotype

Considering S × L interaction, emergence percentage at 10th DAS, dry biomass weight, fresh shoot weight, dry shoot weight, dry root weight and F_v_/F_m_ ratio values were ranged between 0.0 and 26.7%, 1.02 and 1.72 g, 2.14 and 5.60 gr, 0.43 and 0.80 gr, 0.51 and 0.95 g and 0.62 and 0.70, respectively. However, there was no significant difference among the values of these parameters. Emergence percentage values after 11th DAS were significantly different (except at 19th DAS). Compared to the control treatment, emergence percentages at 24th DAS under medium salinity level with Ca contaning salts, under low salinity level with MgCl, under medium and high salinity levels with NaCl, and under low and medium salinity levels with MgSO_4_ salt sources were significantly decreased. Similarly, high salinity level with CaCl_2_ and MgCl_2_, and medium salinity level with NaCl significantly retarded the mean emergence time of III-28 seeds. Shoot height of the III-28 faba bean genotype were significantly decreased, in general, especially under medium (except with CaSO_4_ salt) and high salinity levels with all salt sources. In general, fresh biomass weights were drastically decreased under increasing all salinity levels with CaCl_2_ and MgCl_2_; under medium and high salinity levels with Na containing salts; and under high salinity level with CaSO_4_ and MgSO_4_ salt sources. Also, fresh root weights under increasing all salinity levels with Ca containing salts, and under medium and high salinity levels with MaCl_2_, NaCl and MgSO_4_ salt sources showed profound decreases (Table 5).

**Table 5:** The interaction effects of S × L on III-28 faba bean genotype.

Parameter	Salinity level (dS/m)	Salt source					
		CaCl_2_	MgCl_2_	NaCl	CaSO_4_	MgSO_4_	Na_2_SO_4_
Emergence percentage at 10th DAS	0.0	13.33^x^a^y^	13.33a	13.33a	13.33a	13.33a	13.33a
	1.0	10.00a	13.33a	23.33a	13.33a	20.00a	26.67a
	2.0	0.00a	20.00a	0.00a	10.00a	16.67a	23.33a
	3.0	0.00a	3.33a	0.00a	13.33a	26.67a	26.67a
Emergence percentage at 24th DAS	0.0	90.00abc	90.00abc	90.00abc	90.00abc	90.00abc	90.00abc
	1.0	83.33cde	80.00def	90.00abc	93.33ab	80.00def	86.67bcd
	2.0	80.00def	96.67a	80.00def	76.67ef	80.00def	83.33cde
	3.0	93.33ab	90.00abc	73.33f	86.67bcd	86.67bcd	90.00abc
Mean emergence time (day)	0.0	11.67de	11.67de	11.67de	11.67de	11.67de	11.67de
	1.0	11.96de	12.25cde	12.63bcde	12.16cde	11.71de	11.25e
	2.0	13.14bcd	11.71de	13.84abc	11.48de	11.59de	12.08de
	3.0	15.28a	13.96ab	12.91bcde	11.77de	12.27cde	12.26cde
Shoot height (cm)	0.0	9.40ab	9.40ab	9.40ab	9.40ab	9.40ab	9.40ab
	1.0	8.10c	6.63def	6.98cde	9.48a	8.08c	6.88cde
	2.0	4.79hi	5.55fghi	5.54fghi	8.13bc	6.88cde	6.24defg
	3.0	3.45j	4.32ij	3.54j	7.40cd	5.76efgh	5.11ghi
Fresh biomass weight (g)	0.0	12.05a	12.05a	12.05a	12.05a	12.05a	12.05a
	1.0	9.37bcd	9.52bcd	11.27ab	9.59bcd	10.25abc	10.27abc
	2.0	7.51def	7.03ef	8.11cde	9.89abc	9.99abc	9.16bcd
	3.0	5.91fg	5.97fg	4.87g	9.82bc	8.63cde	9.44bcd
Dry biomass weight (g)	0.0	1.72a	1.72a	1.72a	1.72a	1.72a	1.72a
	1.0	1.35a	1.24a	1.47a	1.38a	1.38a	1.18a
	2.0	1.17a	1.12a	1.11a	1.49a	1.30a	1.10a
	3.0	1.09a	1.02a	1.03a	1.44a	1.23a	1.10a
Fresh shoot weight (g)	0.0	5.00a	5.00a	5.00a	5.00a	5.00a	5.00a
	1.0	4.07a	3.89a	4.75a	5.6a	4.76a	4.44a
	2.0	3.48a	3.28a	3.97a	4.49a	4.39a	3.54a
	3.0	2.25a	2.55a	2.15a	4.39a	3.60a	3.66a
Dry shoot weight (g)	0.0	0.77a	0.77a	0.77a	0.77a	0.77a	0.77a
	1.0	0.53a	0.54a	0.67a	0.80a	0.68a	0.62a
	2.0	0.53a	0.50a	0.55a	0.69a	0.69a	0.57a
	3.0	0.53a	0.50a	0.44a	0.71a	0.63a	0.58a
Fresh root weight (g)	0.0	7.05a	7.05a	7.05a	7.05a	7.05a	7.05a
	1.0	5.30bcde	5.63abc	6.52ab	3.99efg	5.60abc	5.83ab
	2.0	4.03defg	3.75fg	4.14cdefg	5.40bcde	5.49bcd	5.61abc
	3.0	3.65fg	3.41g	2.73g	5.43bcde	5.03bcdef	5.78ab
Dry root weight (g)	0.0	0.95a	0.95a	0.95a	0.95a	0.95a	0.95a
	1.0	0.82a	0.70a	0.80a	0.58a	0.70a	0.56a
	2.0	0.64a	0.62a	0.56a	0.80a	0.61a	0.54a
	3.0	0.66a	0.51a	0.60a	0.73a	0.59a	0.52a
F_v_/F_m_	0.0	0.66a	0.66a	0.66a	0.66a	0.66a	0.66a
	1.0	0.67a	0.66a	0.64a	0.68a	0.69a	0.63a
	2.0	0.67a	0.67a	0.64a	0.66a	0.70a	0.63a
	3.0	0.62a	0.64a	0.65a	0.69a	0.63a	0.62a

^x^Each value is the mean of three replications.

^y^Within salt source and salinity level of each parameter, means followed by the same letter are not significantly different at the 5% level according to Duncan’s multiple range tests.

### Performance of III-29 faba bean genotype

Emergence percentage at 24th DAS (ranged between 80.0 and 100.0%), mean emergence time (ranged between 9.41 and 11.45 day) and dry shoot weight (ranged between 0.34 and 0.63 g) values of III-29 faba bean genotip were not statistically significant under S × L interaction. Throughout the experimental period, only the emergence percentage at 9th, 10th, 11th and 12th DAS showed significant difference. Emergence percentage at 10th DAS was significantly decreased, under high salinity level with CaCl_2_, under medium and high salinity levels with MgCl_2_ and NaCl, and under all increasing salinity levels with CaSO_4_ salt sources. In general, increasing salinity levels with all salt sources (except low salinity level with CaCl_2_ salt) resulted in decreases on shoot heights. Fresh biomass, shoot and root weights exhibited a marked decrease under medium and/or high salinity levels with Cl containing but not with SO_4_ containing salt sources. Decreases on dry biomass weights were observed only under high salinity level with MgCl_2_, and under medium and high salinity levels with NaCl salt sources. Similar to III-1 and III-22, slightly low F_v_/F_m_ ratios were observed under medium and high salinity levels with Na_2_SO_4_ salt source (Table 6).

**Table 6:** The interaction effects of S × L level on III-29 faba bean genotype.

Parameter	Salinity level (dS/m)	Salt source					
		CaCl_2_	MgCl_2_	NaCl	CaSO_4_	MgSO_4_	Na_2_SO_4_
Emergence percentage at 10th DAS	0.0	70.00^x^abc^y^	70.00abc	70.00abc	70.00abc	70.00abc	70.00abc
	1.0	76.67ab	60.00bcdef	63.33bcde	46.67efg	50.00defg	70.00abc
	2.0	63.33bcde	46.67efg	36.67g	50.00defg	70.00abc	56.67cdef
	3.0	33.33g	43.33fg	46.67efg	43.33fg	66.67bcd	86.67a
Emergence percentage at 24th DAS	0.0	90.00a	90.00a	90.00a	90.00a	90.00a	90.00a
	1.0	93.33a	93.33a	96.67a	83.33a	83.33a	93.33a
	2.0	96.67a	90.00a	93.33a	93.33a	96.67a	86.67a
	3.0	93.33a	86.67a	93.33a	80.00a	100.00a	96.67a
Mean emergence time (day)	0.0	10.76a	10.76a	10.76a	10.76a	10.76a	10.76a
	1.0	10.56a	10.94a	10.42a	10.79a	10.30a	10.04a
	2.0	10.39a	10.98a	11.45a	10.24a	10.33a	10.72a
	3.0	11.27a	10.88a	10.98a	10.94a	10.13a	9.41a
Shoot height (cm)	0.0	9.35a	9.35a	9.35a	9.35a	9.35a	9.35a
	1.0	9.08a	7.21bc	7.38bc	6.47cde	7.68b	7.40bc
	2.0	5.99de	5.95de	4.87fg	6.14de	6.21de	5.51ef
	3.0	4.05gh	4.43gh	3.97h	6.66cd	6.07de	5.75def
Fresh biomass weight (g)	0.0	9.34abcd	9.34abcd	9.34abcd	9.34abcd	9.34abcd	9.34abcd
	1.0	11.33a	8.36bcdef	10.27ab	7.91cdefg	8.46bcdef	9.97abc
	2.0	7.34defgh	6.93efgh	6.75fghi	7.48defgh	8.36bcdef	8.89bcde
	3.0	5.65hi	6.26ghi	4.90i	9.11bcd	8.39bcdef	9.09bcd
Dry biomass weight (g)	0.0	1.13abcde	1.13abcde	1.13abcde	1.13abcde	1.13abcde	1.13abcde
	1.0	1.28ab	1.18abcd	1.31a	1.20abc	1.08cdefg	1.08cdefg
	2.0	1.05cdefg	1.08cdefg	0.94fgh	1.11bcdef	1.11bcdef	0.97efgh
	3.0	1.01defg	0.92gh	0.83h	1.30a	1.11bcdef	0.96efgh
Fresh shoot weight (g)	0.0	4.22abcd	4.22abcd	4.22abcd	4.22abcd	4.22abcd	4.22abcd
	1.0	4.99a	4.41abc	4.56ab	3.81bcdef	4.08abcde	4.35abc
	2.0	3.68bcdef	3.55cdefg	3.21efgh	3.56cdefg	3.68bcdef	3.36defgh
	3.0	2.71gh	2.96fgh	2.49h	4.02bcde	3.57cdefg	3.51cdefg
Dry shoot weight (g)	0.0	0.54a	0.54a	0.54a	0.54a	0.54a	0.54a
	1.0	0.63a	0.60a	0.63a	0.59a	0.61a	0.62a
	2.0	0.59a	0.52a	0.47a	0.56a	0.51a	0.55a
	3.0	0.44a	0.43a	0.34a	0.57a	0.58a	0.50a
Fresh root weight (g)	0.0	5.12bcd	5.12bcd	5.12bcd	5.12bcd	5.12bcd	5.12bcd
	1.0	6.34a	3.96def	5.71ab	4.11def	4.38cdef	5.62ab
	2.0	3.66efg	3.37fgh	3.53fg	3.93def	4.69bcde	5.54abc
	3.0	2.93gh	3.30fgh	2.40h	5.08bcd	4.81bcde	5.57abc
Dry root weight (g)	0.0	0.59bcde	0.59bcde	0.59bcde	0.59bcde	0.59bcde	0.59bcde
	1.0	0.65abc	0.57bcde	0.68ab	0.60bcd	0.47def	0.46ef
	2.0	0.47def	0.57bcde	0.48def	0.56bcde	0.59bcde	0.42f
	3.0	0.58bcde	0.49def	0.49def	0.73a	0.53cdef	0.46ef
F_v_/F_m_	0.0	0.66cd	0.66cd	0.66cd	0.66cd	0.66cd	0.66cd
	1.0	0.66cd	0.66cd	0.66cd	0.68bc	0.70ab	0.63de
	2.0	0.66cd	0.68bc	0.66cd	0.67bc	0.66cd	0.60f
	3.0	0.66cd	0.63de	0.65cd	0.72a	0.62ef	0.60f

^x^Each value is the mean of three replications.

^y^Within salt source and salinity level of each parameter, means followed by the same letter are not significantly different at the 5% level according to Duncan’s multiple range tests.

### Performance of USK-1 faba bean genotype

Emergence percentage values of the USK-1 faba bean genotype were ranged from 30.0 to 76.7% at 10th DAS, and from 83.3 to 100.0% at 24th DAS but there was no statistically significant difference among the values of these parameters under S × L interaction. Similarly, mean emergence time (ranged between 10.05 and 11.89 days) did not show a significant difference. Throughout the experimental period, only the emergence percentage at 9th DAS showed significant difference. Compared to control salinity level, shoot heights were significantly decreased especially under medium and high salinity levels with all salt sources. In general, compared to control, significant decreases were obtained for fresh biomass weights under medium and high salinity levels with all Cl containing salt sources and with Na_2_SO_4_ in addition to under high salinity level with MgSO_4_ salt source. Also, fresh shoot weights were negatively affected under high salinity level with CaCl_2_, MgSO_4_ and Na_2_SO_4_, under medium salinity level with MgCl_2_, under low salinity level CaSO_4_, and under medium and high salinity levels with NaCl salt sources. Similarly, medium and high salinity levels with all salt sources except CaSO_4_ and also low salinity level with CaCl_2_ cause significant reduction on fresh root weights. In general, marked decreases on dry biomass weights under medium or high salinity levels with all Cl containing salts and dry shoot and root weights under medium and/or high salinity levels with CaCl_2_ and NaCl salt sources were observed. In contrast, none of the salinity level with SO_4_ containing salt sources have an adverse effect on these dry weight parameters. The F_v_/F_m_ ratio was ranged between 0.58 and 0.72. High salinity levels with CaSO_4_, and low and medium salinity levels with MgSO_4_ resulted in an increase on F_v_/F_m_ ratios whereas the lowest F_v_/F_m_ ratio was observed under high salinity level with Na_2_SO_4_ salt source (Table 7).

**Table 7:** The interaction effects of S × L on USK-1 faba bean genotype.

Parameter	Salinity level (dS/m)	Salt source					
		CaCl_2_	MgCl_2_	NaCl	CaSO_4_	MgSO_4_	Na_2_SO_4_
Emergence percentage at 10th DAS	0.0	60.00^x^a^y^	60.00a	60.00a	60.00a	60.00a	60.00a
	1.0	56.67a	46.67a	73.33a	60.00a	56.67a	76.67a
	2.0	43.33a	40.00a	50.00a	60.00a	60.00a	56.67a
	3.0	40.00a	70.00a	30.00a	53.33a	50.00a	66.67a
Emergence percentage at 24th DAS	0.0	90.00a	90.00a	90.00a	90.00a	90.00a	90.00a
	1.0	93.33a	90.00a	100.00a	90.00a	86.67a	93.33a
	2.0	90.00a	83.33a	93.33a	83.33a	86.67a	96.67a
	3.0	86.67a	93.33a	86.67a	86.67a	96.67a	96.67a
Mean emergence time (day)	0.0	10.73a	10.73a	10.73a	10.73a	10.73a	10.73a
	1.0	11.19a	11.89a	11.00a	10.59a	11.44a	10.05a
	2.0	10.96a	10.30a	10.97a	10.51a	10.97a	10.76a
	3.0	10.96a	10.27a	11.52a	10.66a	11.43a	10.16a
Shoot height (cm)	0.0	10.22a	10.22a	10.22a	10.22a	10.22a	10.22a
	1.0	7.98b	6.01efgh	7.57bc	6.62cdef	7.48bcd	8.05b
	2.0	6.33cdefg	4.95hi	4.91hi	7.25bcde	7.33bcde	6.26cdefgh
	3.0	4.33ij	5.79fgh	3.53j	6.13defgh	6.52cdef	5.09ghi
Fresh biomass weight (g)	0.0	10.12a	10.12a	10.12a	10.12a	10.12a	10.12a
	1.0	8.85abc	8.77abc	9.96a	8.66abc	10.22a	9.81ab
	2.0	7.75cd	6.08ef	6.86def	9.42ab	8.68abc	8.27bcd
	3.0	5.87ef	7.66cd	5.39f	9.64ab	7.25cde	7.71cd
Dry biomass weight (g)	0.0	1.13bcd	1.13bcd	1.13bcd	1.13bcd	1.13bcd	1.13bcd
	1.0	0.91defg	0.89defg	0.97cdef	0.97cdef	1.17bc	1.49a
	2.0	0.87defg	0.91defg	0.72g	1.04cde	1.35ab	1.08cde
	3.0	0.74fg	0.84efg	0.87defg	1.10cd	1.20bc	1.07cde
Fresh shoot weight (g)	0.0	4.37a	4.37a	4.37a	4.37a	4.37a	4.37a
	1.0	4.27ab	3.64abcdef	4.34a	3.31cdefg	4.06abc	4.13abc
	2.0	3.75abcde	2.87efgh	2.94defgh	3.62abcdefg	3.78abcd	3.55abcdefg
	3.0	2.73gf	3.51abcdefg	2.18h	3.59abcdefg	3.40bcdefg	2.81fgh
Dry shoot weight (g)	0.0	0.56abc	0.56abc	0.56abc	0.56abc	0.56abc	0.56abc
	1.0	0.51abcd	0.48abcd	0.53abc	0.46bcd	0.55abc	0.63a
	2.0	0.54abc	0.43cd	0.37d	0.47bcd	0.59ab	0.49abcd
	3.0	0.38d	0.41cd	0.53abc	0.53abc	0.54abc	0.42cd
Fresh root weight (g)	0.0	5.75abc	5.75abc	5.75abc	5.75abc	5.75abc	5.75abc
	1.0	4.58efg	5.13bcde	5.62abcd	5.36abcde	6.16a	5.69abc
	2.0	4.00fghi	3.22hi	3.91ghi	5.81abc	4.89cdef	4.73defg
	3.0	3.14i	4.15fgh	3.20hi	6.06ab	3.85ghi	4.90cdef
Dry root weight (g)	0.0	0.57cdef	0.57cdef	0.57cdef	0.57cdef	0.57cdef	0.57cdef
	1.0	0.40fgh	0.41efgh	0.44efgh	0.52cdefg	0.63bcd	0.86a
	2.0	0.33h	0.47defgh	0.35gh	0.57cdef	0.76ab	0.59cde
	3.0	0.36gh	0.43efgh	0.35gh	0.57cdef	0.66bc	0.65bcd
F_v_/F_m_	0.0	0.65def	0.65def	0.65def	0.65def	0.65def	0.65def
	1.0	0.67bcde	0.68abcd	0.65def	0.66bcdef	0.72a	0.62efg
	2.0	0.64def	0.66bcdef	0.69abcd	0.65def	0.71ab	0.63ef
	3.0	0.68abcd	0.65cde	0.62efg	0.71ab	0.68abcd	0.58g

^x^Each value is the mean of three replications.

^y^Within salt source and salinity level of each parameter, means followed by the same letter are not significantly different at the 5% level according to Duncan’s multiple range tests.

### Comparison of faba bean genotypes under the main effect of salinity levels and salt sources

**Figure 1:**
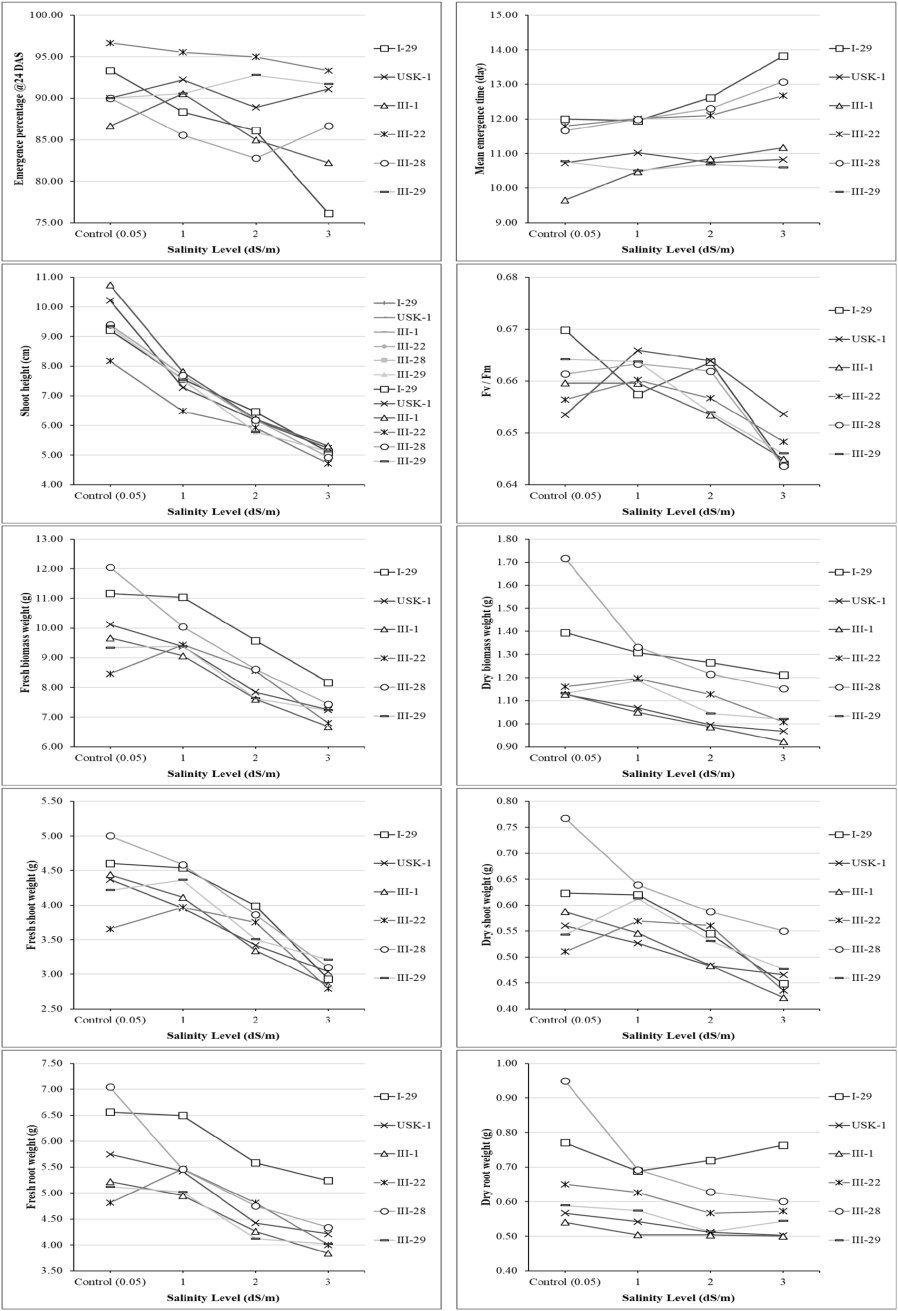
Comparison of faba bean genotypes under the main effect of salinity levels.

Considering all salinity levels, the lowest and the highest values were obtained, in general, from faba bean genotypes of; I-29/III-28 and III-1/III-29 for emergence percentage at 10th DAS, I-29/III-1/III-28 and III-22 for emergence percentage at 24th DAS, III-1/III-29 and I-29/III-22 for mean emergence time, III-22/III-29 and I-29/III-1 for shoot height, III-1/III-22 and I-29/III-28 for fresh biomass weight, USK-1/III-1 and I-29/III-28 for dry biomass weight, USK-1/III-1/III-22 and I-29/III-28/III-29 for fresh shoot weight, USK-1/III-1/III-22 and III28 for dry shoot weight, III-1/III-22/III-29 and I-29/III-28 for fresh root weight, III-1 and I-29/III-28 for dry root weight, and I-29/III-1/III-28 and USK-1 for F_v_/F_m_ ratios (Fig. 1).

**Figure 2:**
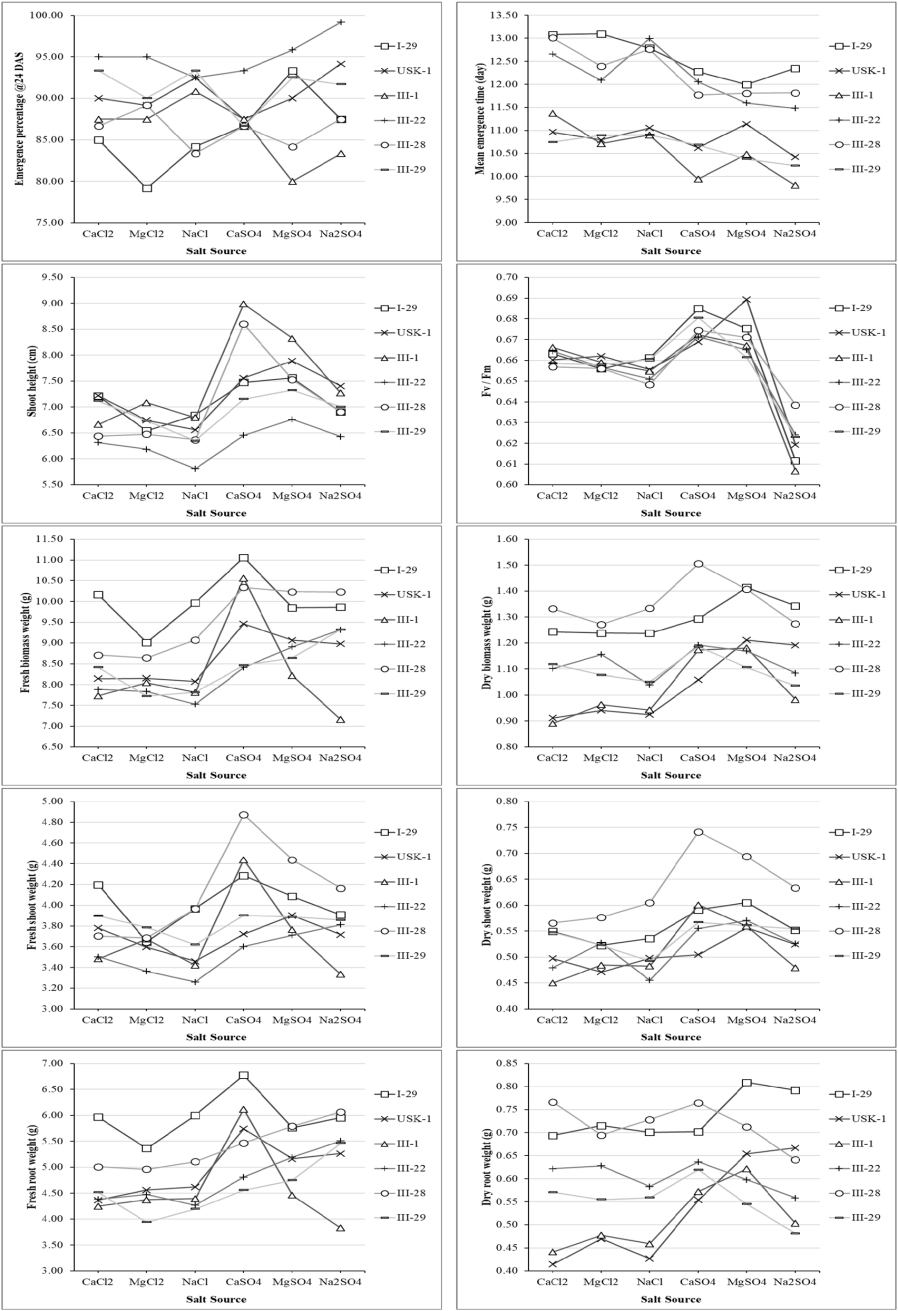
Comparison of faba bean genotypes under the main effect of salinity levels.

Similarly, considering all salt sources, in general, the lowest and the highest values were obtained from I-29/III-22/III-28 and III-1/III-29 for emergence percentage at 10th DAS, I-29/III-1/III-28 and III-22/III-29 for emergence percentage at 24th DAS, III-1/III-29 and I-29/III-22 for mean emergence time, III-22 and I-29/USK-1/III-1 for shoot height, III-1/III-22/III-29 and I-29/III-28 for fresh biomass weight, USK-1/III-1/III-29 and I-29/III-28 for dry biomass weight, III-1/III-22 and I-29/III-28/III-29 for fresh shoot weight, USK-1/III-1/III-22 and III-28 for dry shoot weight, III-1/III-29 and I-29/III-28 for fresh root weight, and USK-1/III-29 and I-29/III-28 for dry root weight values (Fig. 2).

### Principle component analysis (PCA)

**Figure 3:**
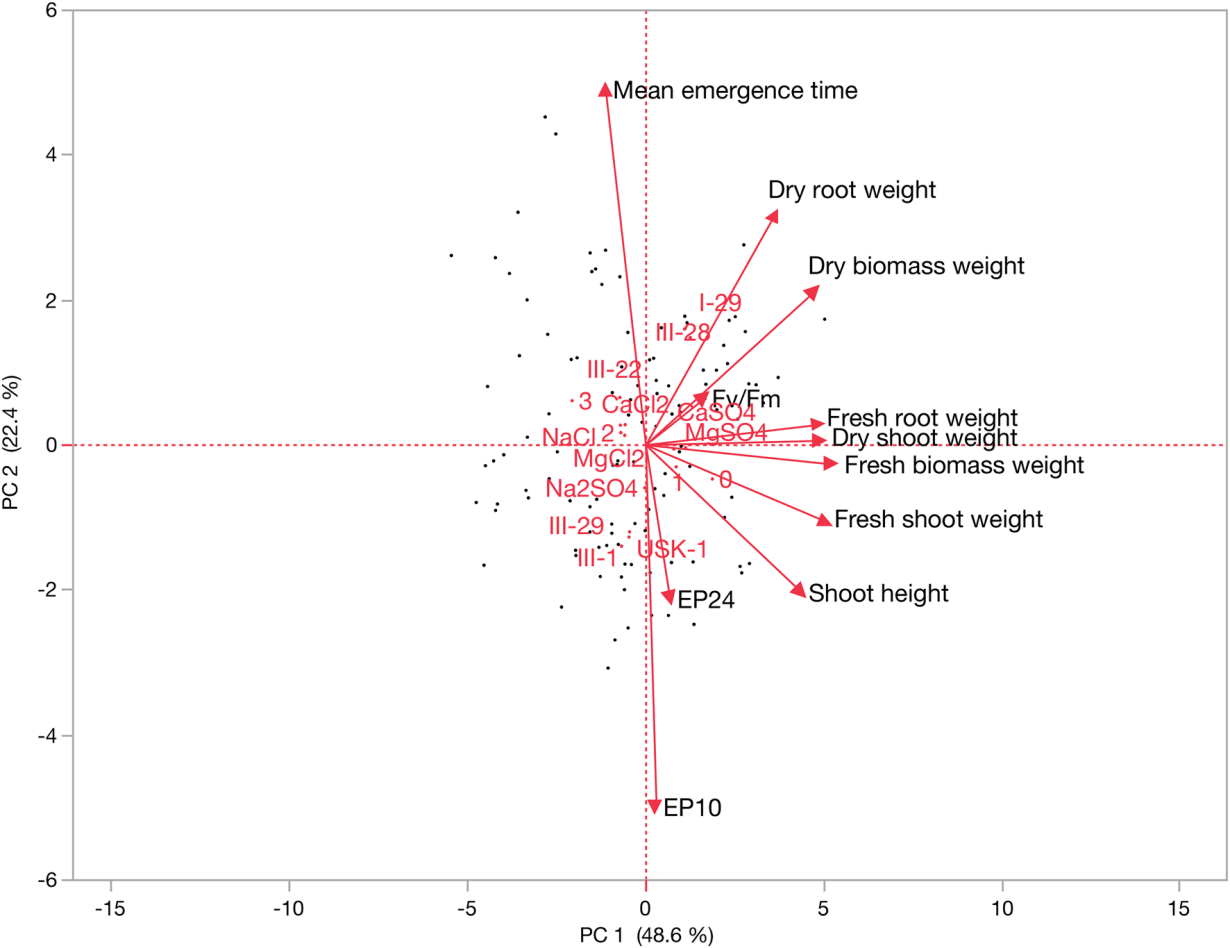
The principal component analysis (PCA) correlation of parameters with different salt sources and faba bean genotypes.

According to PCA, three eigenvalues higher than 1 were found, but the first two components are presented in Fig. 3. A total of 71.0% of the phenotypic variance was explained, with the first component being 48.6% and the second component being 22.4%. The genotype III-22 was the most stable ones. So, this genotype was localized near the origin of the biplot. The high salinity level (3 dS/m) was positioned far from the origin in the biplot indicates that it largely affected the genotypes. The genotypes I-29 and III-28 had a specific respond to salt sources CaSO_4_ and MgSO_4_, while the genotypes III-1 and USK-1 had a specific respond to salt sources CaCl_2_, MgCl_2_ and NaCl. This can be explained by the fact that genotypes react as distinct from to different salt sources (Fig. 3). Also, it was determined that the parameter that best explains the salinity tolerance is F_v_/F_m_. F_v_/F_m_ ratios showed a significant difference for I-29 and III-28 genotypes. Fresh root weight, dry shoot weight, fresh biomass weight, fresh shoot weight and shoot height were found to be positively and significantly correlated with each other and they were negatively correlated with mean emergence time. As expected, these parameters decrease as mean emergence time increases. Dry root weight, dry biomass weight and F_v_/F_m_ parameters were positively correlated with each other. It was determined that I-29 and III-28 genotypes were better than other genotypes, especially in terms of dry root weight and dry biomass weight. While the salt sources that most negatively affected the genotypes were NaCl and Na_2_SO_4_, it was determined that the salt source with less effect was determined for CaSO_4_ (Fig. 3).

### Relationship between investigated parameters

Statistical evaluation results (R and *P* values) of the linear relationships between investigated parameters are presented in Table 8. There were significantly important (*P* < 0.01) positive correlations between parameters of shoot height, fresh biomass weight, dry biomass weight, fresh shoot weight, dry shoot weight, fresh root weight and dry root weight, although, most of the R values seems to be low which indicates a non-linear correlation. Mean emergence time has a negative correlation with all other investigated parameters except dry biomass weight and F_v_/F_m_ ratio (not significant), and dry root weight (a positive correlation). The substantial positive linear correlation was obtained between fresh biomass weight versus fresh root weight (96%) and fresh shoot weight (91%); dry biomass weight versus dry root weight (89%) and dry shoot weight (82%); shoot height versus fresh shoot weight (80%) and fresh shoot weight versus dry shoot weight (80%). The prominent negative relationship was observed between emergence percentage at 10 DAS versus mean emergence time (-75%). However, the relationship between emergence percentage at 10th DAS versus dry shoot weight, fresh root weight and F_v_/F_m_ ratio; emergence percentage at 24th DAS versus dry biomass weight, dry root weight and F_v_/F_m_ ratio; and mean emergence time versus dry biomass weight and F_v_/F_m_ ratio was not statistically significant (Table 8).

**Table 8:** Linear relationship based on correlation coefficient between investigated parameters.

	EP10	EP24	MET	SH	FBW	DBW	FSW	DSW	FRW	DRW
EP24	0.23**									
MET	− 0.75**	− 0.19**								
SH	0.37**	0.17**	− 0.44**							
FBW	0.12**	0.22**	− 0.24**	0.72**						
DBW	− 0.15**	0.09^ns^	− 0.04^ns^	0.52**	0.76**					
FSW	0.21**	0.25**	− 0.34**	0.80**	0.91**	0.68**				
DSW	0.05^ns^	0.17**	− 0.24**	0.59**	0.77**	0.82**	0.80**			
FRW	0.06^ns^	0.19**	− 0.14**	0.60**	0.96**	0.73**	0.75**	0.67**		
DRW	− 0.26**	− 0.01^ns^	0.13**	0.33**	0.55**	0.89**	0.42**	0.47**	0.59**	
F_v_/F_m_	− 0.03^ns^	− 0.05^ns^	− 0.01^ns^	0.20**	0.19**	0.21**	0.23**	0.20**	0.15**	0.16**

*EP10* emergence percentage at 10th DAS, *EP24* emergence percentage at 24th DAS, *MET* mean emergence time, *SH* shoot height, *FBW* fresh biomass weight, *DBW* dry biomass weight, *FSW* fresh shoot weight, *DSW* dry shoot weight, *FRW* fresh root weight, *DRW* dry root weight.

**Significant at 0.01 probability level, *ns* non-significant.

## Discussion

Final emergence percentages were always high (80–100%) for III-22, III-29 and USK-1 faba bean genotypes. Mean emergence time values of I-29, III-1, III-29 and USK-1 faba bean genotypes did not show significant difference under S × L interaction, indicated that the emergence time of these faba bean genotypes was not affected from increasing salinity levels with all salt sources. However, seed emergence was significantly retarded; under high salinity level with CaCl_2_ and under medium and high salinity levels with NaCl salt source for both III-22 and III-28 faba bean genotypes. Similar results have been reported for common bean by Cavalcanti et al.^20^ who studied the effect of water salinity (from 0.7 to 4.7 dS/m), the ionic composition of Na + Mg, and their interaction. They did not observe a significant influence on the percent emergence and emergence speed index, which shows that different genotypes from the same species can have different behaviors.

In general, increasing salinity level regardless of the salt source resulted in decreases in shoot heights of these genotypes. Similarly, it was reported that the emergence and growth of castor bean were more affected by the salinity level than by the cationic composition of the irrigation water^21^. Also, it was stated that shoot height values of common bean genotypes were affected by salinity (NaCl) only at 200 mM^13^ whereas shoot height of pinto bean^22^ and emergence rate, shoot height and leaf area of castor bean^23^ were reduced by increased salinity (NaCl) level. The latter researchers also concluded that the reduction in emergence, plant height, leaf area and chlorophyll a and b was much greater for an increase in salinity from 50 to 100 mM NaCl than that from 0 to 50 mM NaCl.

Significant decreases on these parameters were observed especially under high salinity level with majority of the salt sources. The decreases were more pronuonced with Cl containing salt sources. Dry biomass weight values of III-1, III-22, III-29 and USK-1; dry shoot weight values of III-1, III-22 and USK-1; and dry root weight values of III-29 and USK-1 faba bean genotypes were significantly different under S × L interaction. The dry biomass and shoot weight values, in general, significantly decreased under high salinity level with especially some Cl containing and/or Na_2_SO_4_ salt sources whereas, no harmfull effect was observed at any salinity level with CaSO_4_ and MgSO_4_ salt sources. Del Pilar Cordovilla et al.^24^ reported that shoot and dry root weights of faba been at 25th days after onset of salt treatment were 0.76 and 0.35 g under control and 0.69 g under 100 mM NaCl applications, repectively. Similarly, Helal and Mengel^15^ mentioned that NaCl salinity depresses faba been seedling growth and restricts protein formation, CO_2_ assimilation, and especially the incorporation of photosynthates into the lipid fraction.

It is explained that the photosynthetic performances of higher plants can vary widely according to growth forms, species, organs and stages concerning diverse eco-physiological demands^25^. Similarly, it was reported that exposure to salt did not have any immediate effect on F_v_/F_m_ in the Glycophyte *Arabidopsis* and the Halophyte *Thellungiella* species. However, during the development of salt stress over a 14 d period, this parameter fell in *Arabidopsis* exposed to either 100 or 150 mm NaCl. In *Thellungiella*, no change in F_v_/F_m_ occurred, even at the highest salt concentration^26^. In general, F_v_/F_m_ ratios under medium and high salinity levels with Na_2_SO_4_ salt source were the lowest almost for all faba bean genotypes.

Averaged over all salinity levels or salt sources, the performance order of faba bean genotypes was III-28 ≤ III-22 ≤ I-29 < USK-1 ≤ III-1 ≤ III-29 on emergence percentage at 10th DAS; I-29 ≤ III-1 ≤ III-28 < USK-1 ≤ III-29 < III-22 on emergence percentage at 24th DAS; I-29 < III-28 ≤ III-22 < USK-1 ≤ III-29 ≤ III-1 on mean emergence time; III-22 < III-29 ≤ III-28 ≤ I-29 ≤ USK-1 < III-1 on shoot height; III-1 ≤ III-22 ≤ III-29 ≤ USK-1 < III-28 < I-29 on fresh biomass weight; III-1 ≤ USK-1 < III-29 ≤ III-22 < I-29 < III-28 on dry biomass weight; III-22 < III-1 ≤ USK-1 < III-29 < I-29 ≤ III-28 on fresh shoot weight; USK-1 ≤ III-1 ≤ III-22 < III-29 ≤ I-29 < III-28 on dry shoot weight; III-29 ≤ III-1 < III-22 ≤ USK-1 < III-28 < I-29 on fresh root weight; III-1 ≤ USK-1 < III-29 < III-22 < III-28 ≤ I-29 on dry root weight; and III-1 ≤ III-22 ≤ III-29 ≤ III-28 ≤ I-29 ≤ USK-1 on F_v_/F_m_ ratios. These performance orders clearly indicate that the response of cultivars or genotypes to salinity may differs for different growth parameters. Similarly, it was concluded that the growth responses of two faba cultivars (cv. Eresen 87, cv. Filiz 99) to two levels of NaCl salt was significantly different^16^. They revealed that Filiz 99 is slightly more NaCl resistant cultivar compared to cv. Eresen 87. Large genetic variation of faba bean in tolerance to salinity has also been reported in different studies^27,28^.

Averaged over all faba bean genotypes, the order of salinity level in terms of negative effect was high (3 dS/m) ≥ medium (2 dS/m) > low (1 dS/m) ≥ control (0.05 dS/m) on emergence percentage at 10th and at 24th DAS; high (3 dS/m) > medium (2 dS/m) ≥ low (1 dS/m) ≥ control (0.05 dS/m) on mean emergence time; high (3 dS/m) > medium (2 dS/m) > low (1 dS/m) > control (0.05 dS/m) on shoot height, fresh biomass weight, dry biomass weight and fresh root weight; high (3 dS/m) > medium (2 dS/m) > low (1 dS/m) ≥ control (0.05 dS/m) on fresh shoot weight and dry shoot weight; medium (2 dS/m) ≥ high (3 dS/m) > low (1 dS/m) > control (0.05 dS/m) on dry root weight; and high (3 dS/m) > medium (2 dS/m) ≥ low (1 dS/m) ≥ control (0.05 dS/m) on and F_v_/F_m_ ratio. Abd El-Baki and Mostafa^17^ observed that there was a sharp reduction in fresh and dry mass of shoots and roots of faba bean with increasing NaCl salinity. De Lima et al.^21^ and Nobre et al.^29^ observed that increasing water NaCl salinity (only with NaCl) level caused significant decreases in percent emergence, emergence speed index and plant height values of caster bean. Similarly, for common bean, it was claimed that seedling growth at 200, 250 and 300 mM NaCl were drastically affected with regard to control^30^. In general, growth reduction due to increased salinity illustrates the negative effect of salinity on plants, activated by altered osmotic potentials of salt in the root system that limits the gain of the required amount of water^31,32^.

Similarly, averaged over all faba bean genotypes, the order of salt sources, in terms of negative effects, was CaCl_2_ ≥ NaCl ≥ MgCl_2_ > CaSO_4_ ≥ MgSO_4_ > Na_2_SO_4_ on emergence percentage at 10th DAS; CaSO_4_ ≥ MgCl_2_ ≥ MgSO_4_ ≥ NaCl ≥ CaCl_2_ ≥ Na_2_SO_4_ on emergence percentage at 24th DAS; CaCl_2_ ≥ NaCl ≥ MgCl_2_ > MgSO_4_ ≥ CaSO_4_ ≥ Na_2_SO_4_ on mean emergence time; NaCl ≥ MgCl_2_ > CaCl_2_ ≥ Na_2_SO_4_ > MgSO_4_ ≥ CaSO_4_ on shoot height; MgCl_2_ ≥ NaCl ≥ CaCl_2_ > Na_2_SO_4_ ≥ MgSO_4_ > CaSO_4_ on fresh biomass weight; NaCl ≥ CaCl_2_ ≥ MgCl_2_ > Na_2_SO_4_ > CaSO_4_ ≥ MgSO_4_ on dry biomass weight; NaCl ≥ MgCl_2_ ≥ CaCl_2_ ≥ Na_2_SO_4_ > MgSO_4_ ≥ CaSO_4_ on fresh shoot weight; NaCl ≥ CaCl_2_ ≥ MgCl_2_ ≥ Na_2_SO_4_ > MgSO_4_ ≥ CaSO_4_ on dry shoot weight; MgCl_2_ ≥ CaCl_2_ ≥ NaCl > MgSO_4_ ≥ Na_2_SO_4_ > CaSO_4_ on fresh root weight; NaCl ≥ CaCl_2_ ≥ MgCl_2_ ≥ Na_2_SO_4_ > CaSO_4_ ≥ MgSO_4_ on dry root weight; and Na_2_SO_4_ > NaCl ≥ MgCl_2_ ≥ CaCl_2_ > MgSO_4_ ≥ CaSO_4_ on F_v_/F_m_ ratios. These orders show that, in general, the negative effects of Cl containing salt sources were higher than that of SO_4_ containing salt sources. In a study to investigate the effect of Na-, Cl- and NaCl-treated soil on faba bean, it was concluded that both high Na and high Cl reduced growth of faba bean but plants were more sensitive to Cl than to Na^33^. The researchers also mentioned that reductions in growth and photosynthesis were greater under NaCl stress and the effect was mainly additive. Also, they claim that salinity caused by high concentrations of NaCl can reduce growth by the accumulation of high concentrations of both Na and Cl simultaneously, but the effects of the two ions may differ. It was reported that the emergence speed index and the emergence percentage of castor bean seedlings were significantly affected by the studied types of water salinity^21^. They claimed that the order of the cations in the irrigation water, in terms of negative effects, was Na > Na + Ca > Ca > Na + Ca + Mg > K. In another study, it was concluded that common bean seedling growth decreases in different type of salinity, due to the seedlings being unable to adjust osmotically or due to the toxic effects of Cl, SO_4_, and/or Na^34^. They observed that inhibiting effects of Na_2_SO_4_ on shoot height, fresh biomass and root weight parameters were by 20% stronger than those of NaCl. However, in our experiment negative effects of NaCl on these parameters were somewhat higher than those of Na_2_SO_4_ salt source.

Principal Component Analysis was performed to examine how the genotypes differ in terms of salt sources and salinity levels. It was determined that III-28 and I-29 genotypes were more tolerant to MgSO_4_ and CaSO_4_ salt sources and were better explained by dry root weight, dry biomass weight and F_v_/F_m_ parameters. The III-22 genotype had a late mean emergence time than the others. III-29, III-1 and USK-1 genotypes have more sensitivity to salinity than I-29 and III-28 genotypes with all salt sources. In studies, investigating salinity stress in faba bean, principal component analysis was carried out in limited numbers. However, there are a few studies evaluating common parameters with this study. Rajhi et al.^28^ (2020) tested salinity (NaCl) resistance in six different bean genotypes. It was reported that mass of the fresh root, mass of fresh shoot, mass of total fresh plant, mass of the dry root, length of the root and length of the shoot parameters explained the phenotypic variance according to PCA^28^. In another study using NaCl salt source in faba bean, it was reported that the parameters evaluated by PCA, root dry weight, shoot fresh weight, shoot dry weight, root fresh weight, dry seed weight and fresh seed weight were collected in one group^35^. The distribution of parameters is similar to our study.

Salinity is an ever-present major constraint and a major threat to most crops, particularly in areas with irrigated agriculture. It is quite evident that salt stress also significantly affects legume crops. Suitability of poor quality water as a supplemental source for irrigation depends on the level of salinity and solute concentration in the water and the selected crop. Even genotypes of the same species may demonstrate distinct responses to the amount and kind of salts present in irrigation and/or soil, especially during the emergence and early seedling growth. There is an immense need about the knowledge of genotype response to both salt source and salinity level for higher yield across saline environments. This study showed that faba bean genotypes have different behaviors in terms of response to the increasing salinity levels artificially makeup by using different salt sources indicating that salt response of faba bean is genotype-specific. These results provide support for further studies on the effect of the salt sources with different salinity level on emergence and early seedling growth, but these still need to be tied into a general response of the genotypes during vegetative and reproductive phases under these conditions.

## Methods

### Experimental site and plant material

The experiment was carried out in plastic seed trays at Akdeniz University Experimental Research Area of Agricultural Faculty, Antalya, located in the south-west region of Turkey, latitude of 36° 53′ 15″ North and longitude of 30° 38′ 53″ East and 58 m above the sea level. Seeds of six faba bean genotypes including I-29, III-1, III-22, III-28, III-29 and USK-1 which are widely extended varieties in Turkey were used in this study. Some traits of plant materials were given in Table 9.

**Table 9:** Some morphological traits of genotypes.

Genotypes	Origin	Plant height (cm)	Seed yield (kg/da)	100-Seed weight (gr)
I-29	ICARDA**	45.8	45.0	94.1
III-1	ICARDA	42.8	75.0	106.0
III-22	ICARDA	45.0	50.0	88.4
III-28	ICARDA	38.5	95.0	93.5
III-29	ICARDA	50.3	75.0	80.4
USK-1	ICARDA	*	*	*

**International Center for Agricultural Research in the dry areas, *not evaluated.

Plastic trays, of dimension 50 × 30 × 5 cm (length × width × height) each containing 45 cells (4.5 cm deep, 5.0 and 4.0 cm upper and bottom diameter per cell), were used for seed emergence and seedling development. Soils used in the experiment as a sowing substrate were sieved with a 4 mm screen in order to remove large particles and filled to the cells of each seed tray. The experimental soil used in the experiment had sandy-loam with 54.8% sand, 16.9% silt and 28.3% clay particles with a bulk density of 1.43 g/cm^3^. The original electrical conductivity and pH of the saturated soil paste extract were 0.37 dS/m and 7.63, respectively. Soil water contents at field capacity and permanent wilting point were measured as 23.8 and 9.4%, respectively.

### Experimental design and treatments

The experimental factors were six salt sources (CaCl_2_, MgCl_2_, NaCl, CaSO_4_, MgSO_4_, and Na_2_SO_4_), three irrigation water salinity levels with an electrical conductivity (EC_w_) of 1.0 (low), 2.0 (medium) and 3.0 dS/m (high) in addition to distilled water with an electrical conductivity of 0.05 dS/m without any salt source as a control and six faba bean genotypes (I-29, III-29, III-1, III-22, III-28 and USK-1).

At the beginning of the experiment, the amount of salt required for each salt source to generate the desired electrical conductivity values in irrigation waters was determined in the laboratory. Considering the determined amounts, irrigation waters with targeted three different salinity levels for each salt source were made separately ready for use in the experimental area by using 18 plastic water tanks having 100-l volume capacity.

There were three replications with 30 seeds for each salt source and salinity level combinations of each faba bean genotype. Therefore, a total of 570 ([3 salinity levels × 6 salt sources + 1 control] × 3 replication × 30 seed per replication) seeds were used for each faba bean genotype. On the day of sowing, the seeds of uniform size were selected and sown in growth substrate with one seed per cell at a depth of 10 mm depth. Starting from the sowing, each seed tray was equally watered every day considering the salt source and salinity level treatment combination.

### Analyses and measurements

Starting on the 9th day after sowing (DAS) until the experiment lasted at 24th DAS, the number of emerged seeds at each replication was recorded every 24-h. Emergence percentage (EP) was calculated at daily periods as the percent ratio of the number of emerged seeds to the number of total seeds for each replication. Similarly, mean emergence time (MET) was calculated at the end of the experiment as the time (day) average value of all emerged seeds for each replication^36^.

Before terminating the experiment, four replicate fluorescence measurements for each seedling were realized in the middle part of intact undetached leaf blades by using a fluorometer (PAR-FluorPen FP 110/D). The potential quantum efficiency of photosystem II (PSII) was determined using expressions F_v_/F_m_ = (F_m_ − F_0_)/F_m_), in which F_v_, F_0_ and F_m_ are variable, minimal and maximal levels of chlorophyll fluorescence. This is a relative measurement of the PSII, and can, therefore, be used to measure the performance of photosynthesis within a leaf where an F_v_/F_m_ value near to 0.8 is regarded as healthy in most plants^37^. Reduction in the F_v_/F_m_ ratio indicates that the plant is subjected to biotic and/or abiotic stresses^38^. After these measurements, all seedlings were removed from seed tray holes and cleaned with water to remove soils from their roots. The shoots and roots of seedlings were separated by cutting tissues with a razor blade on connecting lines that could be easily seen with a different color of the stem and root tissues. Then, the distance between the cutting point and tip of the longest leaves was measured as shoot heights and fresh and oven-dried (at 70 °C until a constant value) shoot and root weight of the seedlings were obtained.

### Statement on guidelines

All experimental procedures and field studies on plants/seeds comply with relevant institutional, national, and international guidelines and legislation.

### Data analysis

Data on the emergence, seedling growth variables and F_v_/F_m_ ratio were statistically analyzed using Analysis of Variance (ANOVA) with SPSS 13.0 (SPSS Inc., Chicago, IL). The main effects of the interactions between salt source and irrigation water salinity level on investigated parameters were analyzed by univariate regression using SPSS 13.0. Homogeneity and normality were checked before subjecting data to ANOVA. Unless otherwise noted, all statistical tests were performed at the 0.01 level of significance. If ANOVA reported significant differences, treatment mean differences were separated using by Duncan’s multiple range tests at *P* ≤ 0.05. Principal Component Analysis was performed on JMP Pro 16.0.0 (SAS Institute Inc.). Considering correlation coefficient (R) values, the strengths of the linear relationships between investigated parameters were evaluated as strong (R ≥ 0.8), moderate (0.5 < R < 0.8) and weak (R ≤ 0.5)^39^.

## References

[1] Duc G (1997). Faba bean (*Vicia faba* L.). Field Crops Res..

[2] Łabuda H (2012). Flowering and characteristics of useful traits of some faba bean (*Vicia faba* L. var. major Harz) cultivars and breeding lines. Acta Agrobot..

[3] Haciseferoǧullari H, Gezer I, Bahtiyarca Y, Mengeş HO (2003). Determination of some chemical and physical properties of Sakiz faba bean (*Vicia faba* L. Var. major). J. Food Eng..

[4] FAOSTAT. *Crop Statistics* (2020). http://faostat.fao.org.

[5] Pekşen A, Pekşen E, Artık C (2006). Bazı bakla (*Vicia faba* L.) populasyonlarının bitkisel özellikleri ve taze bakla verimlerinin belirlenmesi (Determination of plant characteristics and green pod yield of some faba bean (*Vicia faba* L.) populations). Anadolu Tarım Bilim. Derg..

[6] TUIK. *Crop Production Statistics (Vegetables)* (Turkish Statistical Institute, 2020). https://biruni.tuik.gov.tr/medas/?kn=92&locale=tr.

[7] Hanson BR, Grattan SR, Fulton A (2006). Agricultural Salinity and Drainage. Division of Agriculture and Natural Resources Publication 3375.

[8] Kurunc A (2020). Effects of salt source and irrigation water salinity on growth, yield and quality parameters of *Stevia rebaudiana* Bertoni. Sci. Hortic. (Amst.).

[9] Fernández-García N, Martínez V, Carvajal M (2004). Effect of salinity on growth, mineral composition, and water relations of grafted tomato plants. J. Plant Nutr. Soil Sci..

[10] Dişli Y (1997). Antalya İli Kale (Demre) ilçesi yer altı sulama suyu kalitesi üzerine bir araştırma (A Research on the Subsurface Irrigation Water Quality Of Kale (Demre) District of Antalya Province).

[11] Maas EV, Hoffman GJ (1977). Crop salt tolerance—Current assessment. J. Irrig. Drain. Div..

[12] Ayers RS, Westcot DW (1985). Water Quality for Agriculture—FAO Irrigation and Drainage Paper 29.

[13] Kouam EB, Ndo SM, Mandou MS, Chotangui AH, Tankou CM (2017). Genotypic variation in tolerance to salinity of common beans cultivated in Western Cameroon as assessed at germination and during early seedling growth. Open Agric..

[14] Nadeem M (2019). Grain legumes and fear of salt stress: Focus on mechanisms and management strategies. Int. J. Mol. Sci..

[15] Helal HM, Mengel K (1981). Interaction between light intensity and NaCl salinity and their effects on growth, CO_2_ assimilation, and photosynthate conversion in young broad beans. Plant Physiol..

[16] Bulut F, Akinci Ş (2010). The effect of salinity on growth and nutrient composition in broad bean (*Vicia faba* L.) seedlings. Fresenius Environ. Bull..

[17] Abd El-Baki GK, Mostafa D (2014). The potentiality of *Trichoderma harzianum* in alleviation the adverse effects of salinity in faba bean plants. Acta Biol. Hung..

[18] Hasanuzzaman M, Nahar K, Fujita M, Ahmad P, Azooz MM, Prasad MNV (2013). Plant response to salt stress and role of exogenous protectants to mitigate salt-induced damages BT—Ecophysiology and responses of plants under salt stress. Ecophysiology and Responses of Plants under Salt Stress.

[19] Cohen BH (2008). Explaining Psychological Statistics.

[20] Cavalcanti MLF (2005). Tolerância da mamoneira BRS 149 à salinidade: Germinação e características de crescimento. Rev. Bras. Eng. Agríc. Ambient..

[21] De Lima GS (2016). Emergence, growth, and flowering of castor beans as a function of the cationic composition of irrigation water. Semin. Agrar..

[22] Gholami A, Sharafi S, Sharafi A, Ghasemi S (2009). Germination of different seed size of pinto bean cultivars as affected by salinity and drought stress. J. Food Agric. Environ..

[23] Guo X, Zhou G, Zhu G, Jiao X (2019). Effects of calcium on emergence and seedling growth of castor bean under salinity stress. Curr. Sci..

[24] Del Pilar Cordovilla M, Ligero F, Lluch C (1999). Effects of NaCl on growth and nitrogen fixation and assimilation of inoculated and KNO_3_ fertilized *Vicia faba* L. and *Pisum sativum* L. plants. Plant Sci..

[25] Osmond CB, Winter K, Ziegler H, Lange OL, Nobel PS, Osmond CB, Ziegler H (1982). Functional significance of different pathways of CO_2_ fixation in photosynthesis BT—Physiological plant ecology II: Water relations and carbon assimilation. Physiological Plant Ecology II.

[26] Stepien P, Johnson GN (2009). Contrasting responses of photosynthesis to salt stress in the glycophyte arabidopsis and the halophyte thellungiella: Role of the plastid terminal oxidase as an alternative electron sink. Plant Physiol..

[27] Tavakkoli E, Paull J, Rengasamy P, McDonald GK (2012). Comparing genotypic variation in faba bean (*Vicia faba* L.) in response to salinity in hydroponic and field experiments. Field Crops Res..

[28] Rajhi I (2020). Photosynthetic and physiological responses of small seeded faba bean genotypes (*Vicia faba* L.) to salinity stress: Identification of a contrasting pair towards salinity. Photosynthetica.

[29] Nobre RG, de Lima GS, Gheyi HR, da Lourenço GS, dos Soares LAA (2013). Emergência, crescimento e produção da mamoneira sob estresse salino e adubação nitrogenada. Rev. Cienc. Agron..

[30] Mena E (2015). Effect of salt stress on seed germination and seedlings growth of *Phaseolus vulgaris* L. Cultiv. Trop..

[31] Mer RK, Prajith PK, Pandya DH, Pandey AN (2000). Effect of salts on germination of seeds and growth of young plants of *Hordeum vulgare*, *Triticum aestivum*, *Cicer arietinum* and *Brassica juncea*. J. Agron. Crop Sci..

[32] Rhoades JD, Kandiah A, Mashali AM (1992). The Use of Saline Waters for Crop Production. FAO Irrigation and Drainage Paper 48.

[33] Tavakkoli E, Rengasamy P, Mcdonald GK (2010). High concentrations of Na^+^ and Cl^−^ ions in soil solution have simultaneous detrimental effects on growth of faba bean under salinity stress. J. Exp. Bot..

[34] Kaymakanova M (2009). Effect of salinity on germination and seed physiology in bean (*Phaseolus vulgaris* L.). Biotechnol. Biotechnol. Equip..

[35] Latef AAA, Hasanuzzaman M, Tahjib-Ul-Arif M (2021). Mitigation of salinity stress by exogenous application of cytokinin in faba bean (*Vicia faba* L.). Not. Bot. Horti Agrobot. Cluj-Napoca.

[36] Ellis RH, Roberts EH (1981). The quantification of ageing and survival in orthodox seeds. Seed Sci. Technol..

[37] Maxwell K, Johnson GN (2000). Chlorophyll fluorescence—A practical guide. J. Exp. Bot..

[38] Baker NR (2008). Chlorophyll fluorescence: A probe of photosynthesis in vivo. Annu. Rev. Plant Biol..

[39] Peck R, Devore J (2012). Statistics: The Exploration & Analysis of Data.

